# Using Vital Signs for the Early Prediction of Necrotizing Enterocolitis in Preterm Neonates with Machine Learning

**DOI:** 10.3390/children11121452

**Published:** 2024-11-28

**Authors:** Rosa Verhoeven, Thijmen Kupers, Celina L. Brunsch, Jan B. F. Hulscher, Elisabeth M. W. Kooi

**Affiliations:** 1 Department of Surgery, Division of Pediatric Surgery, University Medical Center Groningen, University of Groningen, Hanzeplein 1, P.O. Box 30.001, 9700 RB Groningen, The Netherlands; r.verhoeven@umcg.nl (R.V.); j.b.f.hulscher@umcg.nl (J.B.F.H.); 2 Department of Neonatology, Beatrix Children’s Hospital, University Medical Center Groningen, University of Groningen, Hanzeplein 1, P.O. Box 30.001, 9700 RB Groningen, The Netherlandsc.l.brunsch@umcg.nl (C.L.B.); 3 Researchable, Zernikepad 12, 9747 AN Groningen, The Netherlands

**Keywords:** necrotizing enterocolitis, artificial intelligence, machine learning, near-infrared spectroscopy, vital signs, early prediction, preterm neonates

## Abstract

Background/Objectives: Necrotizing enterocolitis (NEC), a devastating neonatal gastrointestinal disease mostly seen in preterm infants, lacks accurate prediction despite known risk factors. This hinders the possibility of applying targeted preventive therapies. This study explores the use of vital signs, including cerebral and splanchnic oxygenation, measured with near-infrared spectroscopy in early NEC prediction. Methods: Several machine learning algorithms were trained on data from very preterm patients (<30 weeks gestational age). Time Series FeatuRe Extraction on the basis of scalable hypothesis tests (TSFRESH) extracted significant features from the vital signs of the first 5 postnatal days. We present the F1-scores and area under the precision-recall curve (AUC-PR) of the models. The contribution of separate vital signs to the selected TSFRESH features was also determined. Results: Among 267 patients, 32 developed NEC Bell’s stage > 1. Using a 1:4 NEC:control ratio, support vector machine and logistic regression predicted NEC better than extreme gradient boosting regarding the F1-score (0.82, 0.82, 0.76, resp., *p* = 0.001) and AUC-PR (0.82, 0.83, 0.77, resp., *p* < 0.001). Splanchnic and cerebral oxygenation contributed most to the prediction (40.1% and 24.8%, resp.). Conclusions: Using vital signs, we predicted NEC in the first 5 postnatal days with an F1-score up to 0.82. Splanchnic and cerebral oxygenation were the most contributing vital predictors. This pioneering effort in early NEC prediction using vital signs underscores the potential for targeted preventive measures and also emphasizes the need for additional data in future studies.

## 1. Introduction

Necrotizing enterocolitis (NEC) is the most lethal gastrointestinal disease seen in neonatal intensive care units (NICUs) [1]. It involves an acute inflammation of the intestines due to an immature intestinal defense system, an aberrant microbiome, and repetitive episodes of systemic hypoxia [2]. The rapid progression of NEC can lead to intestinal necrosis, perforation, and hemodynamic instability [3]. With a mortality rate of 30–50% in advanced cases, NEC is a major cause of neonatal deaths in the preterm population [4,5,6]. The incidence of NEC is inversely related to gestational age and birth weight, primarily affecting very preterm infants (gestational age < 32 weeks) at an incidence of 2–7% [7,8,9]. These numbers are rising as we continue to improve at keeping extremely preterm babies alive [10,11].

The appearance of NEC is staged using the modified Bell’s criteria [12,13]. Bell’s stages IIa-IIIb describe the clinical signs and symptoms for radiologically proven and advanced NEC. Despite several known risk factors such as low gestational age and birth weight, early prediction of which infants will develop NEC IIa or higher remains challenging [14]. Without these methods, the options for targeted preventive measures are limited, especially considering the rapid progression of the disease.

Previous research has indicated that the use of vital signs, in particular cerebral and splanchnic oxygenation measured with near-infrared spectroscopy (NIRS), might allow for early prediction of NEC. These measurements reflect systemic perfusion and maturity of the gastrointestinal tract. Abnormalities in these measurements could, for instance, identify bowel ischemia due to the brain-sparing effect [15,16,17]. Arterial oxygenation might also provide useful information in combination with these measurements. For example, low arterial oxygenation in combination with high splanchnic oxygenation might suggest low metabolism due to necrotizing intestines. Finally, heart rate and respiratory rate might be used to assess the infant’s general condition [13].

However, monitoring and evaluating these data over time can be quite challenging, which is where artificial intelligence (AI) might be able to support physicians. AI has been shown to be able to make fast and increasingly accurate predictions in medicine with the use of big data [18]. In particular, machine learning (ML), a subset of artificial intelligence, involves training systems to identify patterns in datasets and to make decisions or predictions about new data that improve over time.

The current study aims to investigate the use of supervised ML as a predictive tool for NEC, using vital signs collected at a high frequency from the first days after birth. Three ML algorithms (logistic regression, support vector machine, and extreme gradient boosting) were trained on the birth characteristics and features automatically constructed from continuous vital signs of preterm infants admitted to the NICU of the University Medical Center Groningen to predict NEC. We decided to leave out laboratory values and other potential risk factors in order to focus on the always present and unbiased highly frequent vital data. This emphasis on vital signs was intended to create a strong proof of concept that could be seamlessly integrated into existing clinical workflows.

Next to predicting NEC, we investigated which vital signs contributed most to this prediction. Understanding the vital signs most relevant prior to the development of NEC is crucial for early intervention. Developing models capable of supporting physicians in the NICU with the early prediction of NEC can facilitate targeted preventive measures, thereby helping to prevent NEC and reduce NEC-related morbidity and mortality. This study aims to examine the usefulness of vital signs as a foundation for more comprehensive deep learning approaches.

## 2. Materials and Methods

### 2.1. Patient Selection

Due to the retrospective design of this study, the study was considered exempt from Institutional Review Board approval and the need for informed consent. Preterm infants (gestational age < 30 weeks) admitted to our NICU between January 2018 and June 2022 were included in the study. The patients needed to have survived for more than 7 days after birth. Patients who developed NEC within those first 7 days were also excluded to allow us to develop an algorithm that is predictive in nature, rather than diagnostic. By maintaining a minimal 48 h gap between the last included data point and the clinical onset of NEC, we focused on predicting risk based on postnatal factors rather than early-onset cases, which may involve distinct pathophysiological mechanisms such as congenital or perinatal factors. Finally, patients were excluded if their vital signs of the first 5 days after birth were not available due to technical data transfer reasons.

The definitive presence of NEC within the resulting group was determined on the basis of pneumatosis intestinalis and/or portal air on the abdominal X-ray, surgical reports, and pathology reports. Two senior consultants (E.K. and J.H.) independently reviewed all cases. Doubtful cases were discussed until a consensus was reached on whether to include the patient in the NEC or control group. Spontaneous intestinal perforations were excluded based on surgical and/or pathology findings.

Due to the imbalanced nature of the data (more control patients than NEC patients) and the fact that machine learning algorithms are not able to deal well with such an imbalance [19], the control group was randomly downsampled to a 1:4 NEC:control ratio. This ratio was inspired by classic case-control study guidelines, as it is considered a good compromise between achieving statistical power and maintaining a reasonable case-control balance [20,21]. To attain more reproducible results and for the purpose of training the models, this process was repeated 20 times, resulting in 20 distinct patient samples that each represent a unique combination of the full set of NEC patients and a subset of control patients.

### 2.2. Data Preprocessing

The final predictors selected from the databases included birth characteristics and automatically constructed features from the patients’ continuous highly frequent vital signs (Table 1). Birth characteristics included gestational age, birth weight, and sex. The vital signs were measured in the first 5 days after birth and consisted of heart rate, respiratory rate, arterial oxygenation (SpO2; all measured at 1 Hz with Philips monitor IntelliVue MX750, Philips Healthcare, Best, The Netherlands), and cerebral and splanchnic oxygenation (rSO2; measured at 0.2 Hz with NIRS using regional oximeter INVOS 5100c, Medtronic, Mansfield, MA, USA). These data reflect real-life measurements routinely obtained as part of standard care in our NICU. All these vital signs are stored in accordance with our hospital’s safety and privacy standards.

Time Series FeatuRe Extraction on the basis of scalable hypothesis tests (TSFRESH) was used to extract and select significant features from the time series data of the vitals [22]. These constructed features enabled the utilization of machine learning algorithms that do not typically work with raw time series data. While deep learning algorithms excel with raw data, they demand substantial training samples. Due to our limited dataset, we opted for simpler machine learning algorithms instead, which rely on predefined features [23]. Assessing the selected features and their original vital signs helped gauge how much the different vital signs contributed to the prediction.

To derive these features, we first imputed missing values in the vital signs time series through linear interpolation. Subsequently, we downscaled the time series to hourly averages for noise reduction. Using the TSFRESH extraction package, numerous features were derived from the modified time series (e.g., absolute maximum, number of peaks, Fourier coefficients). After feature extraction, we used the TSFRESH selection package to determine the relevance of each feature for the classification to be made (NEC or control) via univariate significance testing. Only statistically significant features were retained using the Benjamini-Hochberg procedure [24], using a *p*-value threshold of 0.05. This process was repeated for all 20 distinct patient samples.

Finally, the data were normalized and prepared for performing a stratified k-fold cross validation of each ML model. This meant that for each of the 20 randomly selected patient samples, the sample was split up into 32 evenly balanced folds, ensuring that each fold contained data of one NEC patient and four control patients. This approach allows the models to be tested on each fold after being trained on the others. Subsequently, the model’s overall performance is calculated as the average performance across all testing folds. This method enhances the generalizability and consistency of results between cross-validations [25]. All preprocessing steps were performed with Python 3.9 [26].

### 2.3. Model Development and Analysis

Characteristics of patients with NEC and control patients were compared in R [27]. Categorical data are presented as percentages and compared using Chi-squared tests. Continuous data are presented as median [interquartile range] and are compared with Mann–Whitney U tests. Administration of probiotics is not compared because we have only recently started using this treatment for the latest patients in our patient selection.

We compared three ML prediction models: logistic regression (LR), support vector machine (SVM), and extreme gradient boosting (XGBoost). These three models represent traditional logistic regression, a classic machine learning algorithm, and an ensemble learning technique, respectively. All models were implemented with scikit-learn v.1.1.2 in Python [26,28].

For each algorithm, F1-scores and precision-recall area under the curve (AUC-PR) are presented, portraying predictive power ranging from zero (no power) to 1 (maximal power). These measures are taken as an alternative to the regular measures of accuracy and receiver operating characteristic-AUC, respectively, which are less suitable for imbalanced datasets. The F1-score is a harmonic balance between precision (“how many patients that were predicted to get NEC by the algorithm did indeed develop NEC?”) and recall (“how many of the patients that actually developed NEC were predicted to get NEC by the algorithm?”). AUC-PR quantifies the precision scores for each recall threshold. F1 and AUC-PR are presented as mean ± SD, as calculated over the 20 patient samples. One-way ANOVA (presented as F(degrees of freedom between algorithms, degrees of freedom within algorithms) = F-statistic, *p*-value) is employed to assess variations in predictive power among the algorithms. In the presence of a significant difference, post-hoc pairwise Tukey HSD comparisons are conducted to identify specifically between which algorithms the difference is observed.

Additionally, we evaluated the contribution of each vital sign to the statistically significant TSFRESH features. The contribution is presented as mean percentage ± SD, as calculated over the 20 patient samples. These analyses were also performed in Python [26].

## 3. Results

### 3.1. Included Patients

**Figure 1:**
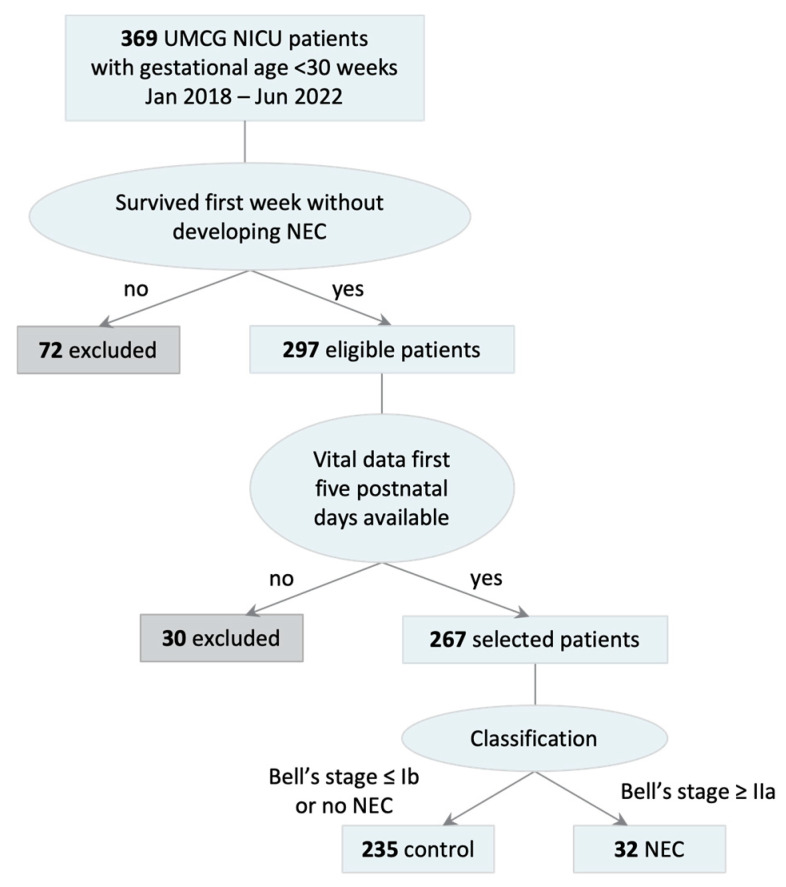
Patient inclusion. Patients with gestational age < 30 weeks, who survived for more than 7 days without NEC, and whose vital signs were available for the first 5 days after birth are included. Patients with Bell’s stage IIa or higher are categorized as NEC.

Patient selection resulted in 267 patients of whom 32 developed NEC at Bell’s stage IIa or higher at least 8 days after birth (Figure 1).

Table 2 summarizes the demographic and clinical characteristics of all patients in this database. This table shows no significant difference in sex between the NEC group and the control group. Infants with NEC had lower gestational age and birth weight than infants in the control group, and more patients with NEC died. There were no differences in administration of antenatal steroids and postnatal antibiotics between the two groups.

### 3.2. Prediction Models

Table 1 shows the percentage of missing and imputed vital signs. All required birth characteristics were known. Most missing data were seen in the splanchnic and cerebral oxygenation data.

The F1-scores and AUC-PR of the prediction models are listed in Table 3. A one-way ANOVA yielded a significant difference in F1-score among the algorithms (F(2,57) = 7.62, *p* = 0.001). Post-hoc comparisons using the Tukey HSD test indicated that the highest mean F1-scores were obtained by LR and SVM, without significant difference (*p* = 0.969). XGBoost had an F1-score lower than both LR (*p* = 0.003) and SVM (*p* = 0.005). A one-way ANOVA also indicated a significant difference in AUC-PR among the algorithms (F(2,57) = 11.88, *p* < 0.001). Again, LR and SVM showed the highest AUC-PR without a significant difference (*p* = 0.974). AUC-PR of XGBoost was lower than in both LR and SVM (both *p* < 0.001).

### 3.3. Vital Sign Contribution

Table 4 shows the average percentage of contribution of the vital signs to the selected TSFRESH features. We observed that several hundreds of TSFRESH features were used for the prediction and that there was notable variability in the selected features among the 20 patient samples. On average, most TSFRESH features have been derived from the splanchnic rSO2, followed by cerebral rSO2. Respiratory rate contributed least to the selected TSFRESH features.

## 4. Discussion

We investigated the use of continuous vital signs from the first days after birth in the early prediction of NEC in preterm infants. SVM and LR predicted NEC with an F1-score up to 0.82. Splanchnic and cerebral oxygenation, though containing missing data, contributed most to the TSFRESH features used in the prediction.

Machine learning models apply various data-fitting strategies, and common practice in artificial intelligence is to compare models for specific tasks [29]. SVM and LR are classical machine learning algorithms that are more often used in medical applications [30]. Despite distinct mathematical foundations, they yielded similar results in our dataset, whereas XGBoost performed less effectively. XGBoost is known for its high predictive power through ensemble learning with decision trees, especially on large tabular datasets [31]. Hence, in the current study, its subpar performance may be attributed to the relatively small dataset used.

Predicting NEC in this study heavily relied on early postnatal vital signs, offering both advantages and drawbacks. If successful, the algorithms could alert physicians of a risk for NEC at a very early stage, unlike other studies that often use biomarkers collected right before or even after NEC diagnosis [32,33,34]. Detecting NEC at a much earlier stage enables preventive targeted measures such as administration of probiotics for at-risk individuals while reducing over-treatment for low-risk patients, minimizing unnecessary side effects [35].

Another advantage of using these early vital signs is that they are readily available as they are constantly measured for all patients with gestational ages below 30 weeks. Meeus et al. [36] used a similar approach, but they combined NEC with late-onset sepsis prediction and only found a median time gain of <10 h. Additionally, unlike other studies [37,38], we decided not to provide the algorithms with any additional laboratory or clinical data, such as exposure to antibiotics or the used feeding method, to keep the prediction as objective and unbiased as possible. This approach also ensures that the model can be implemented in practice with minimal effort from the clinical team. However, future research should focus on increasing the model’s predictive capabilities by complementing vital signs with laboratory and clinical values and examining their additional value to further improve outcomes for at-risk neonates.

Despite the displayed potential of using vital signs, in this retrospective cohort, many data were missing, in particular from splanchnic and cerebral oxygenation. This is the result of local care protocols, such as sensor removal for kangaroo care or not being placed due to limited skin space, as well as technical difficulties resulting in data not being stored temporarily. Consequently, we employed linear interpolation to impute these vital signs, which is only a rough approximation of what actually happened between two data points. While this approach helped to maintain local trends and structures, it also reduced variability, possibly increasing apparent certainty in the data. This could influence the reliability of TSFRESH features, introducing a higher risk of type I errors (false positives). On the other hand, training algorithms on more complete data might also increase prediction certainty. Hence, improving and expanding our oximetry measurements remains a priority for ongoing research efforts. In addition, future attempts may benefit from more sophisticated imputation methods to enhance data quality and reduce potential biases inherent in linear interpolation.

After interpolation, we used TSFRESH for automatic feature extraction and selection from these imputed time series, since machine learning algorithms require predefined features as input. TSFRESH is a Python package that is regularly used to deal with time series data in medical contexts [39,40,41]. It allows for extracting features from the time series and filtering out the ones that are statistically significant for the classification to be made [22]. However, one disadvantage of TSFRESH is the complexity of its computed variables, thereby counteracting the notion of transparency, which is often requested by end-users [42]. For instance, variable names like “rSO2_2__change_quantiles__f_agg_”var”__isabs_False__qh_0.8__ql_0.0” might be challenging to interpret. Hence, even if the models would display important variables that contribute to a patient’s prediction, the user might still consider the models a black box. This contrasts with approaches like HeRO monitoring, which leverages heart rate variability metrics to offer clear insights into sepsis risk and its implications for care [43].

This also makes it more difficult to determine the features’ validity and to interpret what the relatively large contribution of the vital signs to the selected TSFRESH features implies. The data seem to support the findings of previous studies, which showed that splanchnic and cerebral oxygenation might be able to contribute to the early prediction of NEC [44,45]. However, given the missing data and lack of interpretability of the included features, we cannot determine the validity of this assumption yet. Additionally, next to the ambiguity of the TSFRESH features, we also observed a notable variability in selected variables among the 20 patient samples, which suggests that variations in the input lead to different sets of features being considered significant in predicting NEC. This inconsistent performance implies that the selection process is not yet completely robust.

Because of our limited dataset, we had to make several concessions. First, we chose to apply a 1:4 case:control ratio to balance statistical power and manageable class imbalance, in line with established case-control study guidelines. Second, we decided not to filter out patients who had any pathologies that might mimic or interfere with the clinical picture of necrotizing enterocolitis such as sepsis. Although this makes it harder for the algorithms to distinguish between the pathologies, it should result in a more clinically relevant and valuable model. In future studies, as larger and more balanced datasets become available, we aim to explore models that do not rely on predefined case-control ratios and can better differentiate between NEC and other pathologies, further enhancing the generalizability and accuracy of our predictions. Testing these algorithms on external datasets is essential to effectively lead to more robust predictions and consistently reliable outcomes. Ideally, such a dataset should be prospectively collected, as this would address one of the primary limitations of our study, which stems from its retrospective design. Due to our analysis of historical data, we face challenges like missing data and potential selection bias, making it difficult to accurately assess the model’s validity.

Access to such an extensive, prospective database also ignites the potential for the use of deep learning techniques to attain even more productive predictions. Although deep learning techniques are often considered black boxes, they do often outperform traditional machine learning models in the case of large data quantities [46]. One reason for this is that they can make use of the nuances that are present in raw data. Moreover, research is being done to make deep learning models transparent [47,48]. Developing such transparent deep learning algorithms would also allow us to incorporate clinical factors such as antenatal steroids, exposure to antibiotics, and feeding type while mitigating biases and avoiding self-fulfilling prophecies.

Several studies have already attempted to identify or predict NEC using such deep learning techniques. Promising results are shown in studies that make use of abdominal radiographs, infrared images, and microbiome data [49,50]. In particular, Lin et al. [51] used a supervised multiple-instance learning approach, in which data from stool samples were added every day to update the risk score. They concluded that they were able to predict NEC up to 8 days before onset. As also mentioned by them, the addition of highly frequent vital data such as ours might lead to an unbiased, accurate model that could predict NEC even earlier on. Using such an approach would enable more precise predictions of both early and late-onset NEC despite their distinct pathophysiological mechanisms.

In conclusion, we have seen that the use of vital signs in the early prediction of necrotizing enterocolitis is promising but needs further refinement. Particularly, splanchnic and cerebral oxygenation seem to be useful in this prediction. Given these preliminary results, we plan to extend our work with a larger dataset and a multimodal deep learning approach to put together a robust and early prediction of necrotizing enterocolitis.

## Figures and Tables

**Table 1:** Input variables. Overview of the parameters used for the prediction of NEC, including percentages of missing and imputed data for the time series variables.

Variable	Variable Type	Missing Data (%)	Imputed Data (%)
Gestational age	Static	NA	NA
Birth weight	Static	NA	NA
Sex	Static	NA	NA
Heart rate	Time series	9.07	8.94
Respiratory rate	Time series	18.69	15.14
Arterial oxygenation	Time series	13.50	11.84
Cerebral oxygenation	Time series	30.25	27.16
Splanchnic oxygenation	Time series	61.81	50.91

**Table 2:** Patient characteristics.

Group	All (N = 267)	Control (N = 235)	NEC (N = 32)	Difference NEC/Control
Male, N (%)	145 (54.31)	124 (52.77)	21 (65.63)	*p* = 0.171
GA (weeks + days), median (IQR)	27 + 6 (26 + 3 − 29 + 1)	27 + 6 (26 + 4 − 29 + 1)	27 + 2 (26 + 0 − 28 + 1)	*p* = 0.045
BW (grams), median (IQR)	1000 (830–1260)	1010 (840–1280)	878 (749–1038)	*p* < 0.001
NEC onset (days), median (IQR)	-	-	14.50 (10.00–22.25)	
NEC laparotomy, N (%)	-	-	9 (28.13)	
Died, N (%)	18 (6.74)	11 (4.68)	7 (21.88)	*p* =< 0.001
A/N steroids				
N known	228	207	21	
complete (%)	143 (62.28)	130 (62.80)	12 (57.14)	*p* = 0.242
incomplete (%)	57 (25.00)	49 (23.67)	8 (38.10)	
none (%)	29 (12.72)	28 (13.53)	1 (4.76)	
Antibiotics < 72 h P/N				
N known	236	213	23	
yes (%)	169 (71.61)	153 (71.83)	16 (69.57)	*p* = 0.819

**Table 3:** Predictive power for the various ML algorithms. F1-score and area under the precision-recall curve (AUC-PR) of logistic regression (LR), support vector machine (SVM), and extreme gradient boosting (XGBoost) models. F1-score and AUC-PR are presented as mean ± SD calculated over 20 random patient samples.

Algorithm/Measure	F1-Score	AUC-PR
SVM	0.82 ± 0.04	0.82 ± 0.04
LR	0.82 ± 0.05	0.83 ± 0.04
XGBoost	0.76 ± 0.06	0.77 ± 0.04

**Table 4:** Vital sign contribution. Contribution of each vital sign to statistically significant (*p* < 0.05) TSFRESH features. All data are presented as mean contribution ± SD calculated over 20 random patient samples.

Variable	Contribution (%)
Splanchnic oxygenation	40.1 ± 8.2
Cerebral oxygenation	24.8 ± 7.4
Arterial oxygenation	14.5 ± 3.2
Heart rate	12.9 ± 2.4
Respiratory rate	7.6 ± 1.5

## Data Availability

Data supporting reported results can be found at https://doi.org/10.34760/VCQC-XE08.
